# Serious Adverse Events Associated With Hydroxychloroquine Amidst COVID-19 Pandemic: Case Series and Literature Review

**DOI:** 10.7759/cureus.8415

**Published:** 2020-06-02

**Authors:** Ramy Abdelmaseih, Randa Abdelmasih, Mustajab Hasan, Srikanth Tadepalli, Jigar Patel

**Affiliations:** 1 Internal Medicine, Ocala Regional Medical Center/University of Central Florida College of Medicine, Ocala, USA; 2 Cardiology, Ocala Regional Medical Center, Ocala, USA

**Keywords:** hydroxychloroquine, qt prolongation, torsades de pointes, ventricular tachycardia

## Abstract

COVID-19 represents a global health crisis. Several studies are evaluating potential therapies including hydroxychloroquine (HCQ) which is given to patients based on limited observational evidence. However, it can cause serious adverse events. Moreover, recent studies showed no benefits due to HCQ. We present two COVID-19 patients treated with HCQ and had adverse events.

## Introduction

The world is at stake since the first reported case of the COVID-19. The World Health Organization declared COVID-19 outbreak a global pandemic on March 11th, 2020 with more than 120,000 reported cases and more than 4,000 confirmed deaths in over 114 countries. Health organizations and authorities have been fighting shoulder to shoulder to take pre-emptive actions accelerating their efforts to strike the right balance between protecting health, preventing economic and social disruption, and respecting human rights. Clinical trials were launched to investigate potential therapies for COVID-19 including: camostat mesylate, umifenovir, hydroxychloroquine (HCQ), remdesivir, and tocilizumab [1]. However, identifying effective drugs is still a challenge. On the contrary, some of these potential therapies might be more harmful than beneficial. Hence the importance of understanding COVID-19, when it is necessary to treat, and when to do no harm. We present a case series of two COVID-19 patients treated with HCQ and had serious adverse events.

## Case presentation

Case 1

**Figure 1:**
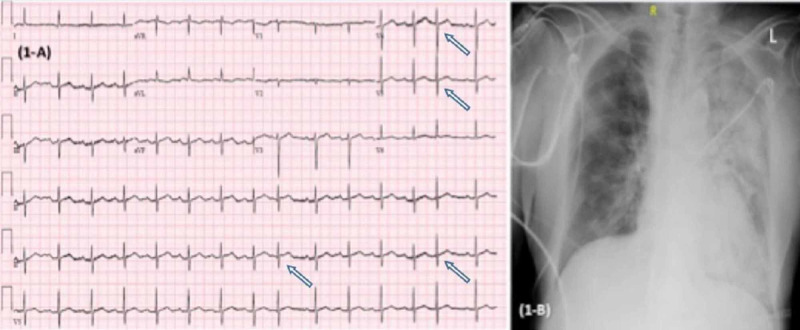
EKG (A) showing sinus rhythm with premature atrial complexes (arrows) with ventricular rate 99 bpm, with nonspecific T wave abnormalities, normal QT/QTc interval 374/457 ms. CXR (B) section showing increased left lung multifocal consolidations concerning pneumonia. EKG, electrocardiogram; CXR, Chest X-ray

**Figure 2:**
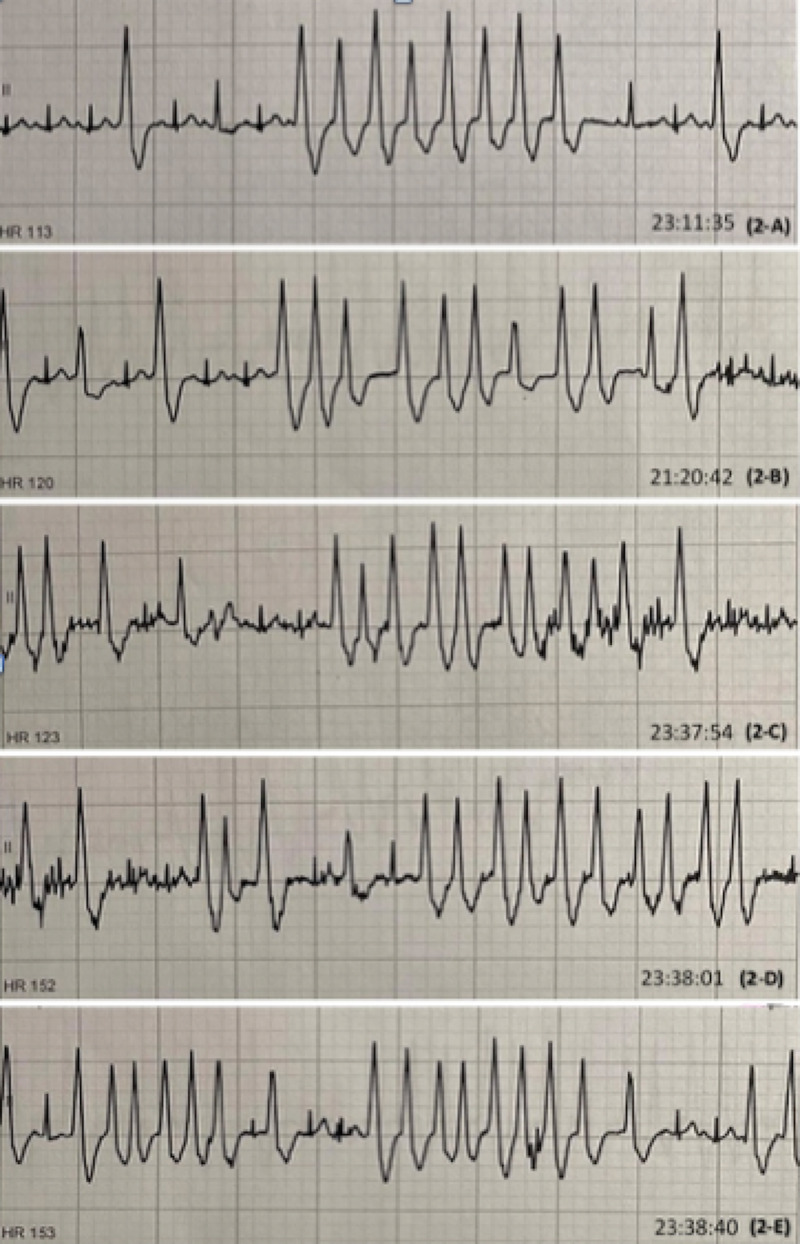
EKG rhythm strips showing multiple episodes of NSVT. (A) showing 8 beat run of NSVT with HR 113, (B) showing 11 beat run of NSVT with HR 120. (C) showing 11 beat run of NSVT with HR 123. (D) showing 10 beat run of NSVT with HR 152. (E) showing 9 beat run of NSVT with HR 153. NSVT, nonsustained ventricular tachycardia

A 75-year-old male with a past medical history of hypertension and diabetes presented to the hospital with worsening shortness of breath, dry cough, fatigue, and fever for three weeks. On presentation, the patient was awake, alert, in moderate respiratory distress, and had left-sided wheezes. He was afebrile with a respiratory rate of 30, oxygen saturation of 95% on nonrebreather mask, blood pressure of 156/81, and heart rate of 86. Chest X-ray (CXR) showed airspace disease involving the left lung (Figure 1). Electrocardiogram showed sinus rhythm with premature atrial complexes. Influenza antigen was negative. Laboratory testing was unremarkable. COVID-19 test was sent and the patient was started on azithromycin and cefepime. The patient had worsening of symptoms and the next day laboratory testing revealed: elevated D-Dimer, ferritin, troponin, lactic acid dehydrogenase, C-reactive protein, white blood cells, and a positive COVID-19 test. The patient was started on HCQ but shortly thereafter, he developed multiple episodes of nonsustained ventricular tachycardia, and HCQ was stopped which led to the resolution of the aforementioned rhythm (Figure 2).

Case 2

**Figure 3:**
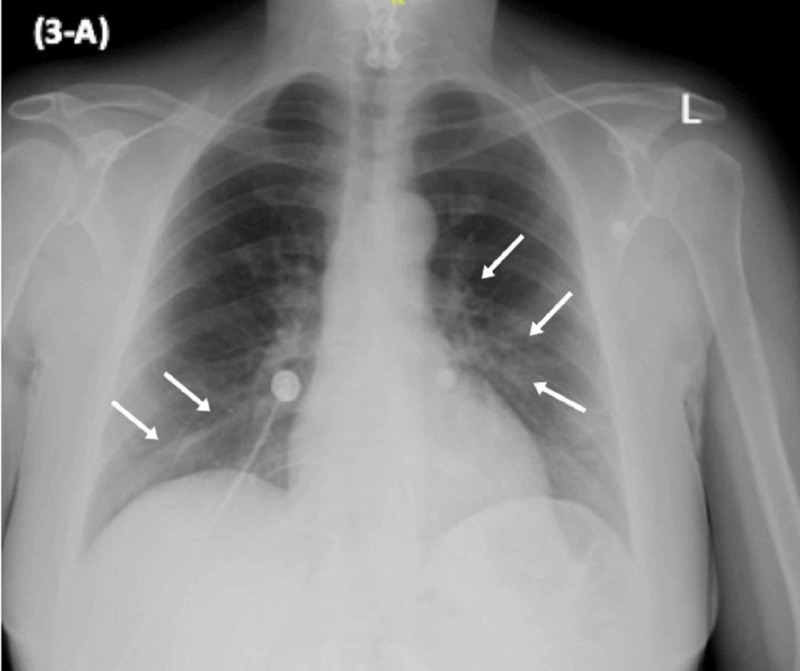
CXR showing bilateral patchy areas of multifocal infiltrate. CXR, Chest X-ray

A 56-year-old COVID-19 positive female was admitted to the hospital for further management of her fever, fatigue, and mild shortness of breath. She denied any gastrointestinal symptoms. On presentation, she was awake, alert with diminished lung sounds bilaterally. She was febrile with a temperature of 100.5°F, respiratory rate of 18 with oxygen saturation 96% on room air, blood pressure of 127/83, and heart rate of 81. CXR showed bilateral patchy areas of multifocal infiltrate (Figure 3). Electrocardiogram showed normal sinus rhythm. Influenza antigen was negative. Laboratory testing including white blood cell count, ferritin, lactic acid dehydrogenase, coagulation profile, liver function tests were within normal range. She was started on HCQ, azithromycin, and cefepime. Soon after initiation of therapy, the patient began complaining of nausea, vomiting, severe abdominal cramps, and unbearable watery diarrhea. HCQ was stopped after two days as per patient’s request and her symptoms improved with supportive care. Ultimately, the patient was discharged home on azithromycin and cefdinir upon improvement and resolution of respiratory symptoms.

## Discussion

COVID-19 pandemic represents the greatest global public health crisis since the pandemic influenza outbreak of 1918. The rapidly expanding knowledge regarding COVID-19 generated a challenge to produce high-quality effective therapies for prevention and treatment. Several potential therapies are being studied including chloroquine and HCQ that interfere with viral entry and endocytosis by inhibiting glycosylation of host receptors, proteolytic processing, and endosomal acidification.

Since the news briefing from China in late February that reported apparent efficacy of chloroquine in treating more than 100 COVID-19 positive cases, HCQ has been given to COVID-19 patients based on limited observational evidence. Moreover, consumers started stockpiling the drug not only for treatment but also for prophylaxis prompted by media reports of possible efficacy. The rationale behind using HCQ and azithromycin is based on a nonrandomized French study that reported improved virologic clearance with combined agents versus standard care [2]. However, the study had several major limitations including: small sample size, dropping patients due to medication intolerance, and concerns of additive cardiotoxicity with combination therapy.

Another Chinese randomized study on 30 COVID-19 positive patients showed no significant difference in median duration from hospitalization day to the viral nucleic acid negative conservation day between the HCQ group and the control group. Also a comparable median time for body temperature normalization, radiological progression, and clinical improvement [3].

Two other important published studies showed no clinical benefit or evidence of a strong antiviral activity. On the contrary, they shed light on the high risk of adverse events including QT prolongation with subsequent fatal cardiac arrhythmias requiring treatment discontinuation especially when combining HCQ to azithromycin [4-5].

Finally, the VA HCQ study -- one of the largest retrospective studies -- evaluated and categorized a total of 368 patients to HCQ arm, HCQ plus azithromycin arm, and no HCQ arm with two primary endpoint outcomes: death and the need for mechanical ventilation [6]. No evidence was found that the use of HCQ, either with or without azithromycin, reduced the need for mechanical ventilation. Surprisingly, an increased overall mortality was identified in patients treated with HCQ alone, enforcing the importance of awaiting the results of ongoing studies before widespread adoption of these drugs. Other studies were prematurely halted because of serious side effects and high death rates with higher HCQ dose [7-8].

Frequent side effects associated with the use of HCQ include: nausea, vomiting, diarrhea, and abdominal cramps. Additionally, it can cause serious cardiac side effects that include arrhythmias due to QT prolongation with associated increased risk of death. HCQ-induced QT prolongation proposed mechanism is through its inhibitory effects on the hyperpolarized cardiac channels causing delayed rates in depolarization leading to decreased heart rates. QT prolongation serves as a surrogate indicator for increased risk of torsades de pointes (TdP) and is associated with increased arrhythmic and nonarrhythmic mortality. Several factors increase the risk of drug-induced QT prolongation including: female sex, structural heart disease, electrolyte disturbance, and hepatic or renal failure [9].

The side effects that we encountered in our case series were arrhythmias causing palpitations and dizziness, and unbearable gastrointestinal symptoms, which resolved upon cessation of HCQ therapy. Patients’ clinical picture improved and they were discharged home without HCQ. They were followed up and reported symptoms resolution. This raises the question around HCQ use: is it really beneficial? Or above all, do no harm.

## Conclusions

COVID-19 presents an extraordinary challenge to identify effective therapies for prevention and treatment. Currently, there is no evidence that any potential therapy has improved disease outcomes. Until further notice, HCQ should be used with extreme caution. All patients should be monitored frequently with electrocardiograms.
